# Chemical Blocking of Zinc Ions in CNS Increases Neuronal Damage Following Traumatic Brain Injury (TBI) in Mice

**DOI:** 10.1371/journal.pone.0010131

**Published:** 2010-04-09

**Authors:** Peter Doering, Meredin Stoltenberg, Milena Penkowa, Jørgen Rungby, Agnete Larsen, Gorm Danscher

**Affiliations:** 1 Institute of Anatomy, Neurobiology, University of Aarhus, Aarhus, Denmark; 2 Section of Neuroprotection, Department of Neuroscience and Pharmacology, Faculty of Health Sciences, The Panum Institute, University of Copenhagen, Copenhagen, Denmark; 3 Department of Pharmacology, University of Aarhus, Aarhus, Denmark; University of North Dakota, United States of America

## Abstract

**Background:**

Traumatic brain injury (TBI) is one of the leading causes of disability and death among young people. Although much is already known about secondary brain damage the full range of brain tissue responses to TBI remains to be elucidated. A population of neurons located in cerebral areas associated with higher cognitive functions harbours a vesicular zinc pool co-localized with glutamate. This zinc enriched pool of synaptic vesicles has been hypothesized to take part in the injurious signalling cascade that follows pathological conditions such as seizures, ischemia and traumatic brain injury. Pathological release of excess zinc ions from pre-synaptic vesicles has been suggested to mediate cell damage/death to postsynaptic neurons.

**Methodology/Principal Findings:**

In order to substantiate the influence of vesicular zinc ions on TBI, we designed a study in which damage and zinc movements were analysed in several different ways. Twenty-four hours after TBI ZnT3-KO mice (mice without vesicular zinc) were compared to littermate Wild Type (WT) mice (mice with vesicular zinc) with regard to histopathology. Furthermore, in order to evaluate a possible neuro-protective dimension of chemical blocking of vesicular zinc, we treated lesioned mice with either DEDTC or selenite. Our study revealed that chemical blocking of vesicular zinc ions, either by chelation with DEDTC or accumulation in zinc-selenium nanocrystals, worsened the effects on the aftermath of TBI in the WT mice by increasing the number of necrotic and apoptotic cells within the first 24 hours after TBI, when compared to those of chemically untreated WT mice.

**Conclusion/Significance:**

ZnT3-KO mice revealed more damage after TBI compared to WT controls. Following treatment with DEDTC or selenium an increase in the number of both dead and apoptotic cells were seen in the controls within the first 24 hours after TBI while the degree of damage in the ZnT3-KO mice remained largely unchanged. Further analyses revealed that the damage development in the two mouse strains was almost identical after either zinc chelation or zinc complexion therapy.

## Introduction

Zinc is found in every cell of our body and is required for processes as diverse as gene expression, DNA synthesis, enzymatic catalysis, hormonal storage, tissue repair, neurotransmission and memory [1], [2]. The majority of zinc ions in the brain (approximately 85%) are intimately bound in proteins, such as zinc finger proteins (DNA-binding proteins), enzymes and metallothioneins, while the rest (approximately 15%) are sequestered in presynaptic vesicles in the terminals of a special subset of neurons, called zinc enriched (ZEN) neurons [3]–[7].

ZEN neurons in the mammalian brain are primarily glutamatergic [8]–[10] and forebrain regions such as hippocampus, amygdala and neocortex are crowded with ZEN terminals [11].

The presynaptic zinc ions are located in a pool of synaptic vesicles. These zinc ions are free or loosely bound and can be chelated by zinc chelators some of which are fluorescent [11], or captured as zinc-selenium nanocrystals and subsequently traced in brain sections at LM and EM levels [12], [13]. The neocortex of rodents displays a dense and highly ordered selenium-AMG staining in the light microscope and reveals the presence of *in vivo* created zinc-selenium nanocrystals in the synaptic vesicles in the electron microscope [13]. The complex patterns of ZEN terminals in different parts of the brain and spinal cord have been described in several papers [14]–[16].

The physiological significance of zinc ions in the ZEN terminals is far from fully understood. It has been suggested that the zinc ions are released into the synaptic clefts [3], [17]–[19] where they act as a neuro-modulating agent on one or more postsynaptic receptors [20], [21].

In 1996 it was suggested that the zinc transporter 3 protein (ZnT3) was responsible for transport of zinc ions into the synaptic vesicles of the ZEN terminals [22]. Later studies confirmed that the ZnT3 protein is abundantly present in the ZEN mossy fibres and that the zinc transporter protein is located to the membranes of the synaptic vesicles and controls the amount of zinc in the synaptic vesicles [23], [24]. ZnT3-KO mice lack the ZnT3 protein and hence these animals are completely void of zinc ions in their ZEN terminals. This finding is supported by the fact that the total zinc levels in the hippocampus and the neocortex have been reported to be reduced by 20% [23], [25]. In harmony with former observations based on a temporary chemical binding of the vesicular zinc pool [26], [27] or a reduced level of vesicular zinc following an adrenally induced loss of zinc ions [28] no major physiological and behavioural changes were found in studies comparing the ZnT3 mouse to the WT littermate. The only recorded important difference was that the ZnT3 mouse tends to be more seizure prone when treated with kainic acid [25], [29].

In addition to influencing neuronal transmission vesicular zinc might contribute to neuronal injury under pathological circumstances where dys-homeostasis of vesicular zinc ions has been advocated to be partaker in the development of the neurodegenerative changes following brain damage [30].

Neuronal damage following TBI [31], ischemia [32], [33] and seizures [34] has been hypothesized to be exacerbated as a result of a dynamic presynaptic release of zinc ions that subsequently transcend into postsynaptic neurons causing cell death.

The present study aims at scrutinizing this hypothesis using ZnT3-KO mice and WT controls, i.e. mice with and without vesicular zinc.

## Materials and Methods

### Animals

The ZnT3-KO and WT mice used were obtained by courtesy of Dr. R. Palmiter.

The animals were kept in plastic cages in a room with a 12-hour light/dark cycle at 21–22°C and 50% humidity. They were fed Altromin No. 1324 (Spezialfutterwerke, Germany) ad libitum and had free access to tap water.

#### Ethics statement

All efforts were made to minimize animal suffering and the number of animals used. The experimental procedures followed were carried out in accordance with the regulations of the animal protection laws of Denmark.

A total of fifty-eight animals were sacrificed during this study (n = 58). The mice selected for chelation therapy were divided into six groups of five (n = 30). The mice selected for *in vivo* selenium autometallography (ZnSe^AMG^) and FluoroJade B staining were divided into four groups of five (n = 20). Eight mice were selected for control sectioning (n = 8).

Temgesic (buprenorphin) was used as an analgesic throughout the procedures.

### The Stereotaxic Cut Lesion

The mice were deeply anaesthetized prior to the operation. The anaesthetic used was a combination of Ketaminol Vet. 50 mg/ml, 0.25 ml Narcoxyl Vet. 20 mg/ml and 3.75 isotonic salt water. 0.1 ml per 10 gram bodyweight of the solution was injected intraperitoneally (IP). Each animal was observed closely for withdrawal reflexes as a test of satisfactory anaesthesia. Then it was placed in a small animal stereotaxic instrument designed to keep the head of the animal securely fixed. The skin above the calvarium was cut mid-sagitally and kept aside by two clips. Under the operation microscope a 1 mm wide and 3 mm long furrow was drilled 2 mm lateral to bregma in the right parietal bone. A scalpel was then mounted in an electronic stereotaxic devise, lowered to the drilled furrow and then further lowered 1 mm, penetrating the neocortex, and gently moved 3 mm caudally. The major advantage of this procedure is that it is highly reproducible and that our model of TBI gives us access to specific target areas with high amounts of ZEN terminals.

The skin above the calvarium was sutured after the operation to avoid introduction of micro-agents into the wound.

### Zinc Ion Capturing

One hour before TBI, the animals chosen for chemical *in situ* capturing of zinc ions were anaesthetized with a Ketaminol/narcoxyl solution and IP injected with either 5 mg per kg bodyweight sodium selenite (Na_2_SeO_3_) or 150 mg per kg bodyweight diethyldithiocarbamate (DEDTC). Sodium selenite and DEDTC were dissolved in deionised water in a concentration of 1 mg sodium selenite per 1 ml distilled water and 15 mg DEDTC per 1 ml distilled water. This low dose of DEDTC (150 mg/kg) only produces a temporary block of the vesicular zinc pattern which within a few hours is re-established, and doses up to 1000 mg/kg DEDTC are tolerated well [35]. Other studies have used doses of 200 mg/kg DEDTC without inducing and without recording apoptosis evaluated with TUNEL stain or evaluated histologically with either H&E or acid fuchsin stains [36], [37].

From studies of retrograde axonal transport of zinc in rats and mice we have found that doses up to 8 mg/kg selenite are tolerated well at a survival time of 24 hours [14], [38], [39], [40]. Thus 5 mg/kg selenite IP is considered a relatively safe dose unable to induce brain damage on its own.

Selenium was chosen as a chemical blocker of loosely bound and free zinc ions because of the long known fact that zinc ions react with selenide ions creating zinc selenide molecules that again accumulate in zinc-selenium nanocrystals [12], [40]. Furthermore, we have previously traced the ZEN terminals to the ultrastructural level applying the ZnSe^AMG^ method, which makes a direct coupling of previous anatomical findings to the findings of the present study possible [41].

DEDTC was chosen because it is a well known, relatively nontoxic chelator with preferences to zinc ions. In 1973 it was found to enable a blockage of all the vesicular zinc ions in the brain [35]. DEDTC is believed to be transformed to Bis(diethyldithiocarbamate)zinc, a diethyldithiocarbamic acid zinc salt. This molecule is not very stable *in vivo* and will within a few hours release the zinc ions/ be metabolized releasing the zinc ions [35].

### Zinc Tracing and FluoroJade B (FJB)

Twenty-four hours after TBI the animals chosen for zinc and FJB staining were re-anaesthetized and sacrificed by transcardial perfusion with a 3% glutaraldehyde solution (GA) in a 0.1% phosphate buffer solution for 3 min. The perfusion pressure was set at 140 mmHg.

### Tissue Processing

The brains selected for TUNEL, caspase-3 or GFAP staining were quickly dissected, dehydrated through graded alcohol, imbedded in paraffin, and cut in series of 3 µm sections.

Brains selected for FJB staining (cryo-sectioning) were post-fixed for at least one hour in a paraformaldehyde (PFA) solution. The newly dissected brains were placed in a 30% solution of sucrose until they sank to the bottom of the jar. The brains were then frozen with CO_2_ for a period of 2 min. Thereafter the tissues were placed in a cryostat and allowed to increase in temperature to −17°C. 30-µm-thick sections were cut in 5 series, placed on glass-slides and stained with FJB according to the protocol (*vide infra*) or were counterstained with a 0.1% aqueous toluidine blue solution, dehydrated in alcohol to xylene, and ultimately embedded in DEPEX and covered with a cover-glass.

### Autometallography (AMG)

The AMG developer consists of a) 60 ml gum arabic solution (Bidinger, Aarhus, DK), b) 10 ml sodium citrate ebuVer (23.5 g sodium citrate (Merck 6448, VWR,DK) to 100 ml distilled water), c) 15 ml reducing agent (0.85 g hydroquinone (Merck 4610, VWR, DK) dissolved in 15 ml distilled water at 40°C), and d) 15 ml of a solution containing silver ions (0.12 g silver lactate (Fluka 85210 supplied by Sigma-Aldrich, Vallensbæk, DK) in 15 ml distilled water at 40°C). Mix solution a (60 ml), solution b (10 ml), and solution c (15 ml) carefully in a 100 ml beaker. Add solution d (15 ml) immediately before use and mix thoroughly. The AMG development takes place in a water bath at 26°C for 60–70 min under a dark hood. Development is stopped by replacing the developer with a 5% thiosulphate solution for 10 min. Finally, the tissue sections/slices are rinsed several times in distilled water [13], [40].

### Tissue Slicing

Tissue slices to be analysed for zinc staining at the light microscopical level were treated in accordance with the following two procedures, respectively:

1. For light microscopical analyses of thick sections the slices were placed in a 30% solution of sucrose until they sank to the bottom of the glass. The slices were then frozen with CO_2_, placed in a cryostat, and allowed a temperature fall to −17°C. After AMG development (see below), the sections were counterstained with a 0.1% solution of aqueous Toluidine blue (pH 4.0), dehydrated in ascending concentrations of alcohol and xylene, embedded in DePex and covered with a cover-glass.
2. For light microscopical analysis of zinc staining in semi-thin sections the slices were cut on a vibratome and the resulting 100-µm-thick sections were developed in AMG. The areas to be analysed were cut out, placed in osmium tetroxide (1% in phosphate buVer for 30 min) and embedded in Epon. From these Epon blocks 3-µm-thick sections were cut and counterstained with toluidine blue.

### FluoroJade B (FJB) Staining

The FJB staining solution consists of: 10 mg of a stock solution of FJB dye powder in 100 ml of distilled water (0.01%). To make up 100 ml staining solution, 4 ml of the stock solution is added to 96 ml of 0.1% of acetic acid vehicle. This results in a final dye concentration of 0.0004%.

The tissues for FJB staining were cryo-proctected with sucrose 20–30% for 24 hours, frozen with CO_2_ and cut in 30 µm sections on a cryostat. The sections were dried on a slide warmer at 50°C for at least half an hour. The slides were then immersed in a solution containing 1% sodium hydroxide in 80% alcohol (20 ml of 5% NaOH added to 80 ml of absolute alcohol) after which they were placed in 70% alcohol for 2 min and distilled water for another 2 min. They were then treated with a 0.02% potassium-permanganate solution for 10 min while being gently shaken on a “shaker” table. Finally the slides were yet again rinsed in distilled water for 2 min.

The slides were placed in a 0.0004% FJB solution for 20 min while being gently shaken, and subsequently rinsed for 1 min in each of three distilled water washes. Excess water was removed by briefly draining the slides vertically on a paper towel. They were then placed on a slide warmer, set at 50°C, for 5–10 min (until dry), and cleared by immersion in xylene for approximately 1 min before being cover-slipped with DPX, a non-aqueous, non-fluorescent plastic mounting media [42].

### Systemic Selenium and DEDTC Treatment

Twenty-four hours after TBI (and 25 hours after IP injections of selenite and DEDTC, respectively), the animals were re-anaesthetized and sacrificed by transcardial perfusion with 4% PFA in a 0.1% phosphate buffer solution for 3 min. The perfusion pressure was set at 140 mmHg.

### *In situ* Detection of Apoptosis

*In situ* detection of DNA fragmentation (TUNEL) Terminal deoxynucleotidyl transferase (TdT) - mediated deoxyuridine triphosphate (dUTP)-biotin nick end labelling (TUNEL) was performed using the Fragment End Labeling (FragEL) Detection Kit (Calbiochem, USA, code QIA33). The FragEL kit contains all the materials used below and each step was performed according to the manufacturer's recommendations. Rehydrated sections were incubated with 20 µg/ml proteinase K for 20 min to strip off nuclear proteins. After immersion in an equilibration buffer for 20 min, the sections were incubated with TdT and biotin-labeled deoxynucleotids (dNTP-biotin) in a humified chamber at 37°C for 90 min. This was followed by a wash buffer and the stop solution for 5 min at room temperature to stop the reaction. After washing in TBS and incubation in blocking buffer for 10 min the sections were incubated with peroxidase-streptavidin for 30 min, and finally DAB was used as chromogen. The sections were counterstained with methyl-green. Negative control sections were treated similarly, but incubated in the absence of TdT enzyme, dNTP-biotin or peroxidasestreptavidin.

### Caspase-3

The paraffin embedded sections were rehydrated according to standard procedures, after which they underwent heat-induced epitope retrieval and blocking as previously described in detail [43], [44]. Sections were then incubated overnight at 4°C with the following primary antibody: Rabbit anti-caspase-3 (activated/cleaved caspase-3 as a marker of apoptosis) diluted 1∶50 (Cell Signaling Techn. Inc., USA, cat. no.: 9661). On the second day the primary antibody was detected by incubating the sections for 30 min at room temperature with a biotinylated secondary antibody: anti-rabbit IgG (Sigma-Aldrich, cat. no.: 3275, diluted 1∶400).

The staining was then visualized using a streptavidin-biotin-peroxidase complex (StreptABComplex/HRP, Dako, Glostrup, DK, code K377) and a tyramide signal amplification (TSA)-kit (NEN, Life Science Products, Waltham, MA, USA, code NEL700A), which were applied following the manufacturer's recommendations. Then, immunoreactions were visualized by using 0.015% H_2_O_2_ in 3,3-diaminobenzidine-tetrahydrochloride (DAB)/TBS for 10 min at room temperature.

### *In situ* Detection of Reactive Gliosis

#### GFAP

The sections were incubated overnight at 4°C with the following primary antibody: Rabbit anti-GFAP (a marker of astrocytes) diluted 1∶250 (Dako, DK, cat. no.: Z334). The primary antibody was detected using biotinylated secondary antibodies (incubation for 30 min at room temperature), followed by streptavidin-biotin-peroxidase complexing (StreptABComplex/HRP, Dako, DK, code K377) and tyramide signal ampliWcation (TSA)-kit (NEN, Life Science Products, USA, code NEL700A), according to the manufacturer's recommendations. Finally, the immunoreactions were visualized using DAB as chromogen.

In all experiments, the extent of non-specific binding of antiserum was evaluated by omitting the primary antibody step. Results were considered only if these controls were negative.

#### Cell Counts

Positively stained cells were defined as cells with positive staining of the soma except in the case of TUNEL staining, where the apoptotic cells were defined as those with nuclear staining (nuclear TUNEL).

The cell counts were performed on five mice per group by the same investigator, who was blinded to the animals' identity and treatment. The quantifications were used for statistical comparisons.

Five sections per animal were collected from the lesioned hemisphere and counted. Every third section down through the lesion was collected, starting randomly on slides 1–3 until a total of 5 sections were obtained from each animal using the Cavalieri principle. A frame was superimposed on the border of the lesion tract ranging into the lesioned area where the damaged cells were counted.

All pictures used for counting procedures were taken in one session and all counting procedures were performed on light microscopic photographs taken using the ×20 objective on a Carl Zeiss Vision light microscope equipped with Carl Zeiss Imager.Z1 and with an AxioCam MRc5 from AxioLab. Fixed settings and conditions were used to ensure proper comparison of the images.

### Statistical Analysis

The average cell counts obtained from the Zinc/ FJB groups were compared using Students t-test. The groups used for chelation treatment and multiple comparisons were analysed using analysis of variance (ANOVA). All values are given as means ± SEM (with a 95% confidence interval (ci)), and the significance level was set at P<0.05.

## Results

### Autometallographic Analysis

**Figure 1:**
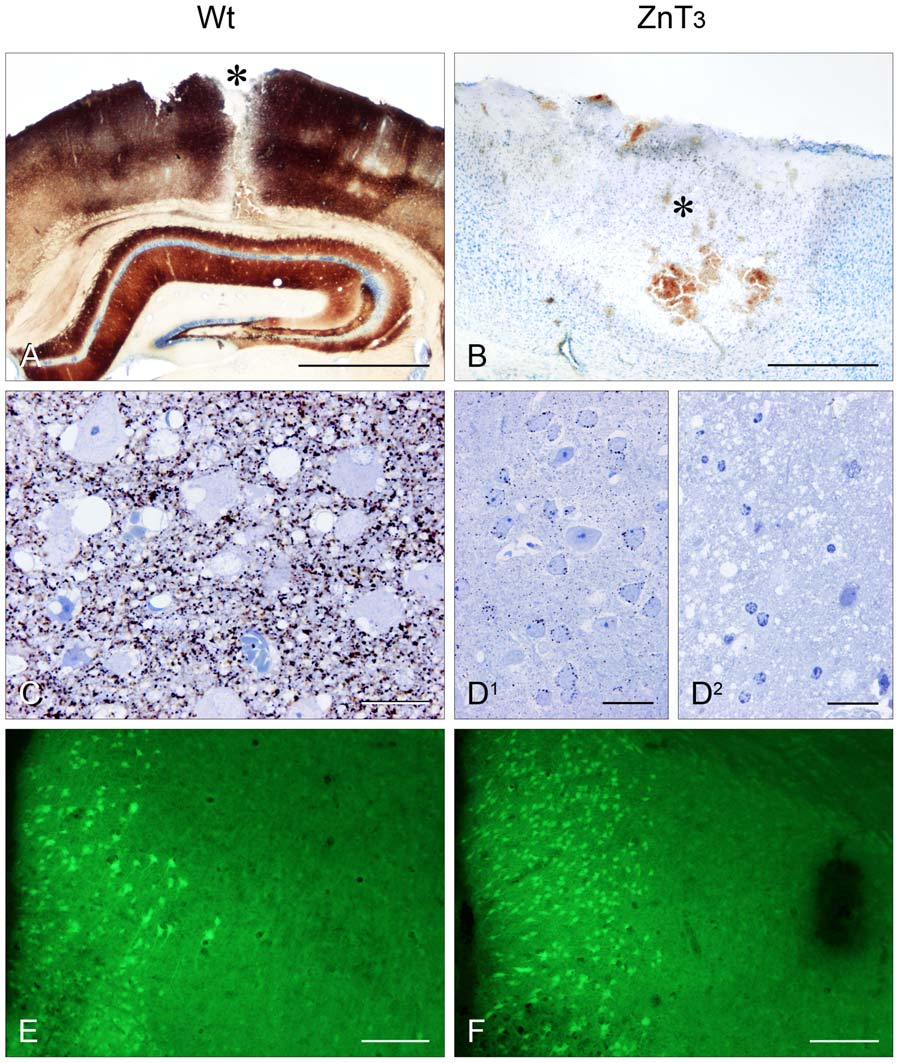
Tracing of zinc using the ZnSe^AMG^ method. (A) Cryo-section: Wild Type mouse 24 hours after TBI. Asterisks denote lesion tract; a distinct layering of the neocortex is noticeable. Closest to the lesion tract no AMG development is possible because all cells are severely damaged. This is shown as a little tint of white on both sides of the lesion tract. Scale bar: 1 mm. (B) Cryo-section: ZnT3-KO mouse 24 hours after TBI. Asterisks denote lesion. In the periphery of the lesion a number of ZnSe nanocrystals containing neurons are just visible. These somata marked neurons border the area between morphologically damaged tissue and morphologically intact tissue. (C) Semi-thin section: Wild Type mouse, a close-up of what is seen in A. There is a distinct neuropil stain; the tissue is oedematous with bleeding. The neurons are distorted, with vacuolation of their cytoplasm and some with eccentrically placed, pycnotic nuclei; none of the neuronal cell-bodies contain any ZnSe nanocrystals. Scale bar: 30 µm. (D_1–2_) Semi-thin sections: ZnT3-KO mouse. In the periphery of the lesion some neurons containing ZnSe nanocrystals in their somata can be seen. Going towards the lesion tract most cells are heavily distorted with condensed, eccentrically placed nuclei. The tissue is severely damaged with massive oedema. Scale bar: 30 µm. Tracing of dead and dying neurons with Fluorojade B (FJB). (E) Cryo-section: Wild Type mouse stained with FJB; fluorescent neurons border the lesion tract. Scale bar: 300 µm. (F) Cryo-section: ZnT3-KO mouse stained with FJB; numerous neurons line the lesion tract. Scale bar: 300 µm.

In the ZnT3-KO mice the ZnSe^AMG^ method revealed numerous neurons with silver enhanced zinc nanocrystals in their somata. After 24 hours the loaded neuronal somata were all found in the periphery of the lesion, marking the transition between TBI influenced neocortex and morphologically intact brain tissue (Figure 1B, D ^1^-D^2^).

Opposite to this characteristic ZnT3-KO TBI AMG pattern the WT control mice were completely void of stained neuronal somata although clear signs of morphological cell damage were conspicuous. LM analysis revealed that the ZnSe nanocrystals were confined only to the neuropil (see Figure 1A, C [41]).

### Cell Death/FluoroJade B Neurons

Neuronal counting of FJB positive neurons and statistical analysis show that the ZnT3-KO mice have significantly more dead or dying neurons evaluated after 24 hours after TBI (Figure 1E, F). Students t-test revealed this to be a significant difference with P<0.05 (see Table 1).

**Table 1:** Students t-test.

TBI	n = 10 mean	95% ci
WT	n = 5 570.6	[348.16 793.04]
ZnT3	n = 5 983.0	[664.20 1301.80]
Diff	n = 5 −412.4	[−769.58 −55.22]*

FluoroJade B staining of cryo sections.

* Indicates statistically significant difference, *p*<0.05.

### Apoptotic Cell Death/TUNEL

**Figure 2:**
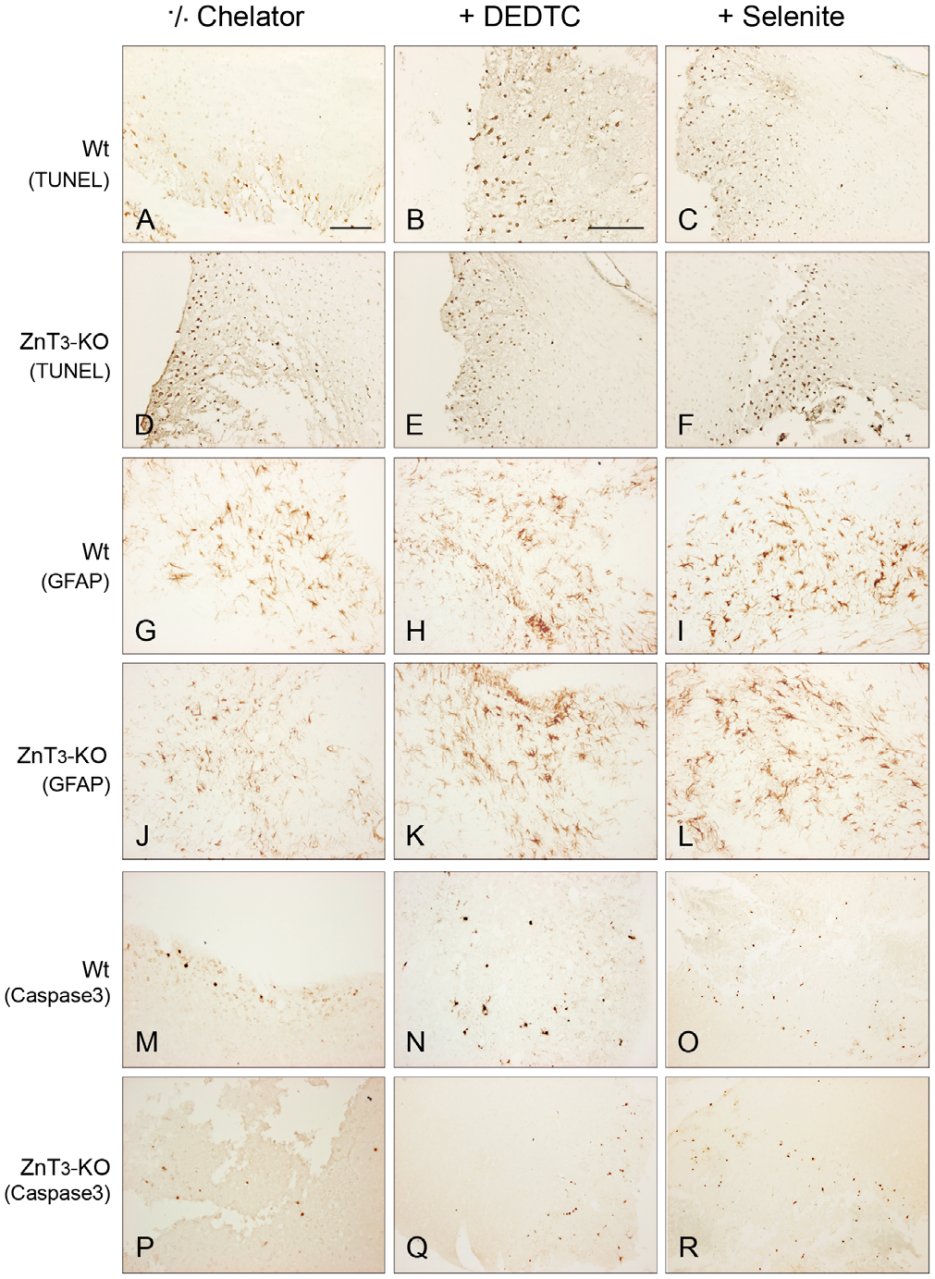
Evaluation of apoptosis and reactive gliosis. The rows illustrate TUNEL stains in (A-F), GFAP stains in (G-L) and Caspase 3 stains in (M-R). All sections are paraffin sections 24 hours after TBI: (A) Wild Type mouse, not chelator treated. (B) Wild Type mouse treated with DEDTC 1 hour before TBI. An increased number of apoptotic neurons are seen. (C) Wild Type mouse treated with selenite 1 hour before TBI. Even more apoptotic neurons are lining the lesion tract. (D) ZnT3-KO mouse not chelator treated. Numerous apoptotic neurons are seen. (E) ZnT3-KO treated with DEDTC 1 hour before TBI. Still a number of apoptotic neurons are seen. (F) ZnT3-KO mouse treated with selenite prior to TBI. No major changes compared to the other ZnT3-KO sections are observed. (G) Wild-type mouse not chelator treated; some reactive cells are seen in the area. (H) Wild Type mouse treated with DEDTC 1 hour before TBI. No major changes are observed. (I) Wild Type mouse treated with selenite 1 hour before TBI. No major changes are observed. (J) ZnT3-KO mouse not chelator treated; some activated glia are seen. (K) ZnT3-KO mouse DEDTC treated 1 hour before TBI. The gliosis remains unchanged. (L) ZnT3-KO mouse selenite treated 1 hour before TBI. A massive increase in the reactive gliosis is seen with big amoeboid cells and increased staining intensity. (M) Wild Type mouse. Sparse Caspase 3 positive cells are seen near the lesion tract. (N) Wild Type mouse treated with DEDTC before TBI. More caspase 3 positive neurons are seen in the proximity of the lesion tract. (O) Wild Type mouse treated with selenite before TBI; a clear increase in the number of caspase 3 positive neurons is seen near the lesion. (P) ZnT3-KO mouse. Sparse Caspase 3 positive cells are seen near the lesion tract. (Q) ZnT3-KO mouse, treated with DEDTC before TBI. No significant increase in the number of Caspase 3 positive cells is observed. (R) ZnT3-KO mouse treated with selenite before TBI. A major increase in the number of positive cells is observed in the entire lesioned area. Scale bar: 300 µm.

In the groups not treated with DEDTC the TUNEL stains also reveal a significant difference in the number of apoptotic neurons between the WT and the ZnT3-KO mice, the latter having the majority of damaged neurons (Figure 2A, D). After chelation therapy the pattern of damage in the two stains resembles one another (Figure 2B, E & C, F) and the WT controls exhibit a significantly increased number of apoptotic neurons compared to WT animals not treated with DEDTC or selenite (compare Figure 2B, C to A). Table 2 summarizes the TUNEL results:

**Table 2:** TUNEL; the group o46–50 has significantly more apoptotic neurons than o41–45, this difference equalizes after chelator application [o51–55;o56–60], [o61–65;o66–70].

TBI	n = 30 mean	95% ci
WT o41–45	n = 5 241.2	[164.01 318.39]
ZnT3 o46–50	n = 5 478.8	[401.61 555.99]
WT + DEDTC o51–55	n = 5 532.2	[454.81 609.19]
ZnT3 + DEDTC o56–60	n = 5 590.4	[513.21 667.59]
WT + selenite o61–65	n = 5 588.3	[510.81 665.19]
ZnT3 + selenite o66–70	n = 5 465.8	[388.61 542.99]

### Astrocytes/GFAP

Activated glial cells were counted 24 hours after TBI. The data reveal that the selenite treated ZnT3-KO mice have a higher degree of reactive gliosis compared to the controls. This is made evident by the significant increase in the number of counted astroglia in the o66–70 group (compare Figure 2L to G, J). Table 3 summarizes our GFAP findings:

**Table 3:** GFAP; only the group o66–70 contains more GFAP positive cells although a tendency towards the cells becoming more GFAP positive after chelation therapy is noted.

TBI	n = 30 mean	95% ci
WT o41–45	n = 5 60.16	[51.00 69.31]
ZnT3 o46–50	n = 5 74.96	[65.81 84.11]
WT + DEDTC o51–55	n = 5 54.33	[45.18 63.48]
ZnT3 + DEDTC o56–60	n = 5 70.14	[60.99 79.30]
WT + selenite o61–65	n = 5 59.36	[50.21 68.51]
ZnT3 + selenite o66–70	n = 5 92.79	[83.64 101.94]

### Apoptotic Cell Death/Caspase-3

The caspase 3 stain showed an increased amount of neurons undergoing programmed cell death after selenite treatment (Figure 2O, R). This is also a tendency seen in group o51–55 and group o56–60 where DEDTC is applied as chelator, even though this effect is not significant (compare Figure 2N,Q to M, P). Table 4 summarizes these results:

**Table 4:** Caspase 3; the chelator treated groups o61–65 and o66–70 contains significantly more apoptotic neurons than the remaining groups.

TBI	n = 30 mean	95% ci
WT o41–45	n = 5 17.14	[7.83 26.45]
ZnT3 o46–50	n = 5 22.70	[13.39 32.01]
WT + DEDTC o51–55	n = 5 25.44	[16.13 34.75]
ZnT3 + DEDTC o56–60	n = 5 33.96	[24.64 43.27]
WT + selenite o61–65	n = 5 41.96	[32.65 51.27]
ZnT3 + selenite o66–70	n = 5 86.48	[77.17 95.80]

### Chemical Binding of Zinc in Un-injured Mice, Control Sections

Twenty-four hours before transcardial perfusion, animals chosen for control sectioning were anaesthetized with a Ketaminol/narcoxyl solution and subsequently treated IP with either 5 mg per kg bodyweight sodium selenite (Na_2_SeO_3_) or 150 mg per kg bodyweight diethyldithiocarbamate (DEDTC).

**Figure 3:**
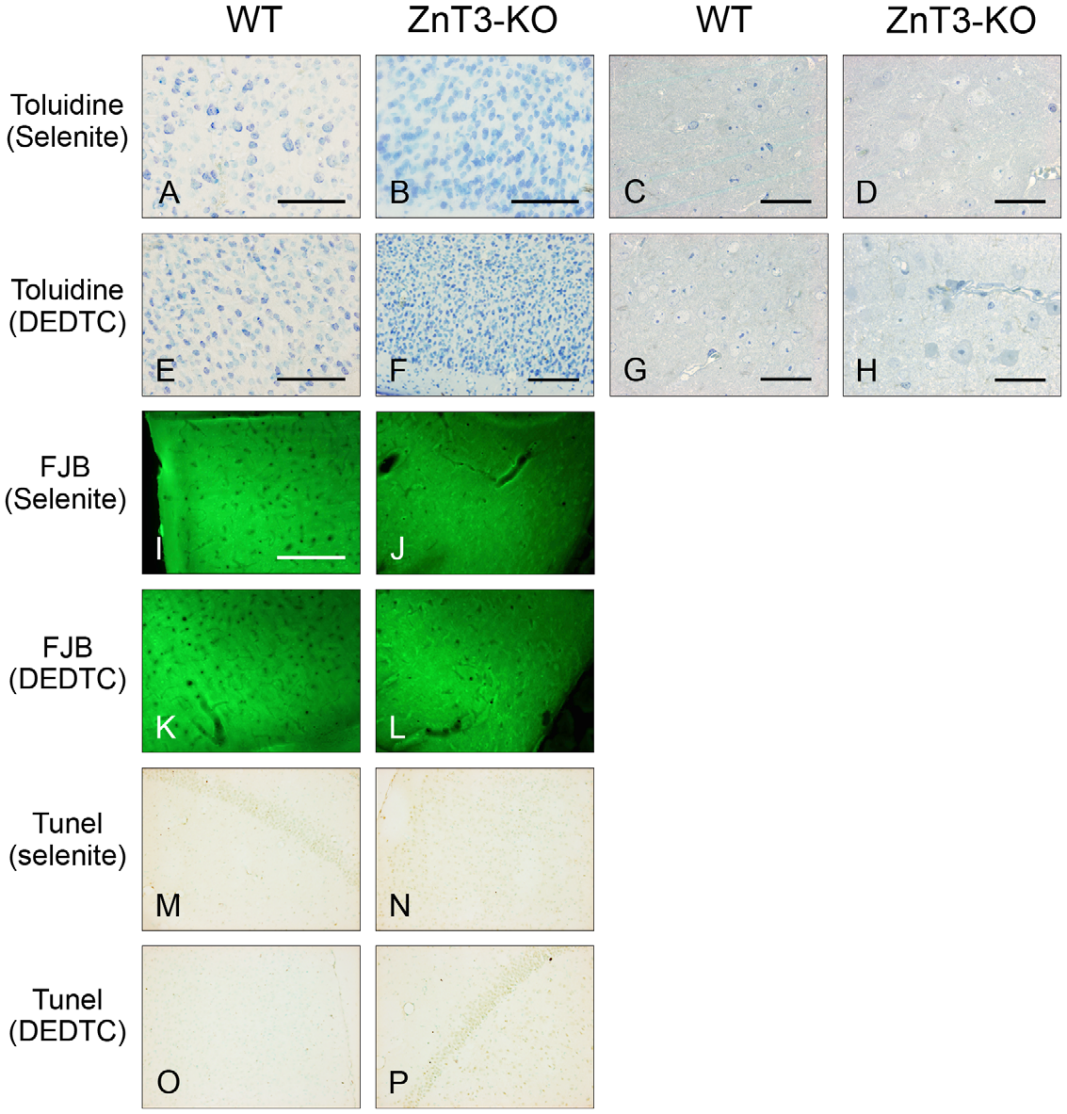
Control sections. Sections are toluidine stained in (A-H). Both the WT mice and The ZnT3-KO mice display a normal morphology 24 hours after respectively DEDTC and selenite treatment. The tissue is without oedema and the cells are all intact with normal configuration. A, B, E, F are Cryo-sections 30 µm thick. C, D, G, H are semi-thin sections 3 µm thick. No major differences between the ZnT3-KO and the WT mice are noticeable. Scale bar A,B: 100 µm. Scale bar E: 200 µm. Scale bar F: 300 µm. Scale bar C, D, G, H: 30 µm. Control sections, FJB stained (I-L). Sections I, J are pretreated with DEDTC and sections K, L are pretreated with selenite. All sections are FJB negative. Control sections, TUNEL stained (M-P). Sections M, N are pretreated with DEDTC and sections O, P are pretreated with selenite. All sections are TUNEL negative. M, P depict part of the hippocampus formation and N, O depict all the six neocortical layers. Scale bar I-P: 200 µm.

The tissue was further processed for either morphological evaluation (Toluidine stain) or for FJB or TUNEL stain. All sections were negative for FJB and TUNEL staining and displayed a normal morphology. There were no observable differences between the WT and the ZnT3-KO mice (See Figure 3).

## Discussion

The finding that TBI exposed neurons of ZnT3-KO mice accumulate zinc ions in their somata and reveal significantly more damaged neurons by the FluoroJade B staining suggests that zinc ions, in complete contrast to what has been suggested until now, have protective qualities to the TBI aftermath. This interpretation was strengthened by the finding that the WT controls after TBI did not accumulate zinc ions in their neuronal somata, although the surrounding neuropil was teeming with ZEN terminals and further that the amount of dead neurons was significantly lower in the WT controls.

This notion was further supported by results from chemical binding of zinc ions. We found that although the ZnT3-KO mice had initially more damaged neurons, as demonstrated by FluoroJade B and TUNEL stains this difference was equalized after chemical binding of the free or loosely bound zinc ions in the ZEN neurons. Further support was gained by the fact that no statistical difference was found in the number of apoptotic neurons between the ZnT3-KO and WT mice exposed to DEDTC and selenium. The observation that the longer period of chemical blocking of zinc ions in zinc-selenide nanocrystals (as opposed to the shorter living zinc-DEDTC molecules) resulted in an increase in the number of GFAP positive cells in the ZnT3-KO mice suggests that the effects of zinc ions might be far more multifarious than hitherto envisaged and possibly that zinc ions are an important part of controlling post TBI inflammation.

The AMG staining seen in the neuronal somata in a zone around the lesion in ZnT3-KO mice must have an origin in the loosely bound or free zinc ions that are dynamically present in all tissues. It can also be deduced that the somata staining does not result from axonal transport when looking at the control group.

The fact that the neurons in the lesion borders of the WT mice were void of zinc ions in their somata 24 hours after TBI is most likely due to the axons being disabled by the nearby cut lesion blocking the normal retrograde transport of vesicular zinc from the terminals. This transport can be traced in undamaged/ normal animals subjected to an IP or IC injection of a sodium selenite/ sodium selenide solution. 6–24 hours after the treatment, ZEN neuronal somata in the whole CNS (injected IP), or ZEN neurons projecting to the injection site (if injected IC into the brain or spinal cord), will be filled with AMG enhanced zinc-selenium nanocrystals. [7], [12], [13], [14], [18], [39], [45]. The only explanation for this lack of transport in a zone around the TBI lesion is therefore that the retrograde axonal transport of zinc ions has been compromised. A likely explanation of the stained somata in the TBI-ZnT3-KO mice might be that the removal of the gene for ZnT3 has in some way changed/influenced the composition of zinc transporters that populate the membranes of ZnT3-KO ZEN neurons. Anatomical studies of TBI have shown that mice with a functioning ZnT3 protein respond to TBI by increasing their terminal content of zinc in a period of 0–8 hours after TBI and that the ZnT3-KO mice exhibit a number of somata marked neurons in the same period of time [41], [46]. We speculate that it is this difference in the neuronal handling/ processing of zinc ions that is responsible for the increased number of damaged neurons in the ZnT3-KO mice. This implies that having a functioning ZnT3 protein is an integral part of reducing brain damage after TBI.

For the last couple of decades it has been suggested that vesicular zinc contributes directly to neuronal damage following insults as diverse as seizure, ischemia and TBI [28], [33], [34]. Other studies have implied that vesicular zinc 1) is not the causative agent of neuronal damage [29], [47], [48], 2) that it excerpts a protective effect [49], and 3) that zinc chelation provides a short-term neuroprotection (evaluated histologically), but fails to improve long-term functional outcome [50].

However, the results obtained in recent studies concerning zinc chelation have been somewhat mixed. Studies using the antibiotic/zinc chelator Clioquinol have revealed an association with this zinc chelating agent and transient global amnesia and neurodegenerative disorders [51]. In related areas of neurobiology, studies on Alzheimer diseased brains have shown a rather deleterious effect when removing zinc from the diet, with a subsequently increased plaque load especially in the neocortex which contains high amounts of ZEN neurons [52].

The fact that genetic (ZnT3-KO mice) or chemical removal (binding) of vesicular zinc increases post TBI cell death could rely on the pro-oxidative effect of zinc removal/ zinc deficiency [53], thereby generating more oxidative stress, or on a direct pro-excitatory effect of zinc deficiency [54]. The lack of effect of zinc binding on the non-injured brain could also reflect the possibility that the main function of the vesicular zinc pool is not to be found during standard physiological conditions, but perhaps rather under pathological situations. Studies of seizures have shown that pre-treatment of mice with DEDTC renders them susceptible to otherwise sub convulsive doses of kainite acid [36], [37] and that the ZnT3-KO mice also have an increased susceptibility to seizures [29] suggesting that vesicular zinc during seizures acts as a neuro-protective agent. We speculate that the function of vesicular zinc in the TBI aftermath may resemble the above scheduled.

Furthermore, low zinc concentration in cells has been associated with pro-apoptotic mechanisms and increased cell death [55], whereas increased zinc levels appear to be anti-apoptotic by reducing oxidative stress [53], [54]. In relation to neurodegenerative disorders, zinc deficiency has been shown to increase the plaque load in a model of Alzheimer's disease, supposedly also due to increased oxidative stress [52].

The fact that TBI on ZnT3-KO mice initially causes more damaged neurons than on the WT mice, and that this difference equalizes after chemical blocking of the vesicular zinc, strongly suggests that vesicular zinc is not the aetiological agent causing neurological damage as suggested by earlier studies [28], [33], [34], [56], [57] and that the vesicular zinc ions have other functions than neuro-degenerative ones in the TBI aftermath.

We hope that the present data may help to clarify the role of zinc ions in the aftermath of brain damage.
